# Riboflavin deficiency induces a significant change in proteomic profiles in HepG2 cells

**DOI:** 10.1038/srep45861

**Published:** 2017-04-03

**Authors:** Zhonghao Xin, Lingling Pu, Weina Gao, Yawen Wang, Jingyu Wei, Tala Shi, Zhanxin Yao, Changjiang Guo

**Affiliations:** 1 Institute of Health and Environmental Medicine, Tianjin, 300050, China; 2 School of Public Health, Guangxi Medical University, Nanning, 530021, China

## Abstract

Riboflavin deficiency is widespread in many regions over the world, especially in underdeveloped countries. In this study, we investigated the effects of riboflavin deficiency on protein expression profiles in HepG2 cells in order to provide molecular information for the abnormalities induced by riboflavin deficiency. HepG2 cells were cultured in media containing different concentrations of riboflavin. Changes of cell viability and apoptosis were assessed. A comparative proteomic analysis was performed using a label-free shotgun method with LC–MS/MS to investigate the global changes of proteomic profiles in response to riboflavin deficiency. Immunoblotting test was used to validate the results of proteomic approach. The cell viability and apoptosis tests showed that riboflavin was vital in maintaining the cytoactivity of HepG2 cells. The label-free proteomic analysis revealed that a total of 37 proteins showing differential expression (±2 fold, p < 0.05) were identified after riboflavin deficiency. Bioinformatics analysis indicated that the riboflavin deficiency caused an up-regulation of Parkinson’s disease pathway, steroid catabolism, endoplasmic reticulum stress and apoptotic process, while the fatty acid metabolism, tricarboxylic citrate cycle, oxidative phosphorylation and iron metabolism were down-regulated. These findings provide a molecular basis for the elucidation of the effects caused by riboflavin deficiency.

Riboflavin (vitamin B_2_) is one of the B vitamins and acts as a precursor of flavin mononucleotide (FMN) and flavin adenine dinucleotide (FAD), which serve as coenzymes for numerous enzymatic reactions and perform key metabolic functions by mediating the transfer of electrons in biological oxidation-reduction reaction^1^. It has been demonstrated that riboflavin is associated with energy production, antioxidant protection, and homocysteine metabolism. The mitochondrial β-oxidation was severely compromised in response to riboflavin deficiency, because several flavin-containing enzymes, such as acyl-CoA dehydrogenase, work in the β-oxidation^2^. Since FAD is a coenzyme for glutathione reductase, riboflavin also plays a role in glutathione synthesis^3^. Besides, riboflavin is also a coenzyme for methylenetetrahydrofolate reductase (MTHFR) that functions critically in homocysteine metabolism, especially in individuals with the MTHFR 677 TT genotype^4^.

Riboflavin deficiency is prevalent in many regions over the world, particularly in underdeveloped countries with a low intake of dairy products and meats^5^,^6^,^7^,^8^. In the clinical conditions, patients with certain types of cancers and congenital heart disease, as well as those with excessive alcohol intake, are at a greater risk of riboflavin deficiency^9^. The poor riboflavin status leads to a variety of clinical abnormalities, including growth retardation, sore throat, cheilosis, anemia, renal damage, neurodegeneration, and possibly even cancers^1^,^10^. Clinical symptoms of riboflavin deficiency are rarely seen in the developed countries, but the subclinical stage of deficiency characterized by a change in biochemical indices, can be found in a significant portion of the populations^1^,^6^,^11^. In spite of a large body of evidences showing that poor riboflavin status interferes with various metabolic processes, the mechanisms of these abnormalities have not been well elucidated so far yet.

HepG2 cells are prone to developing riboflavin deficiency and have been used as a cellular model in several studies^12^,^13^. In the present study, HepG2 cells were cultured in a riboflavin deficient medium. The cell viability, apoptosis and glutathione reductase activity were measured to confirm the development of riboflavin deficiency. Thereafter, a comparative label-free LC–MS/MS analysis was performed to investigate the changes of proteomic profiles in HepG2 cells in response to riboflavin deficiency. The objective of this study is to further provide information for the effects of riboflavin deficiency and the underlying mechanisms using a proteomic approach.

## Results

### Changes of cell viability, apoptosis and glutathione reductase activity after riboflavin deficiency

**Figure 1:**
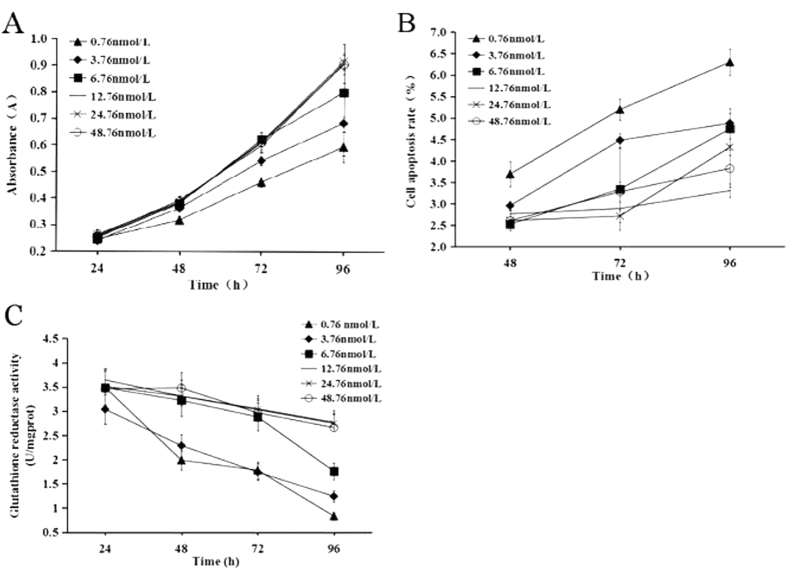
Effect of riboflavin on cell viability, apoptosis and glutathione reductase activity. The riboflavin adequate group (12.76 nmol/L) was defined as the control group. **(A)** cell viability. **(B**) cell apoptosis. **(C**) glutathione reductase activity.

As indicated in Fig. 1a, the cell viability was significantly reduced when the cells were cultured in the riboflavin deficient (0.76, 3.76, 6.76 nmol/L) media compared to the cells cultured in the medium containing 12.76 nmol/L riboflavin (P < 0.05). No significant difference in cell viability was found among the 12.76, 24.76, 48.76 nmol/L riboflavin groups. The changing trend of cell apoptosis was similar to that of the cell viability in response to different riboflavin concentrations (Fig. 1b). Apparently, the concentration of riboflavin in the medium should be lower than 12.76 nmol/L in order to induce a significant change in cell viability and apoptosis. Meanwhile, the activity of glutathione reductase, a sensitive marker for riboflavin status, was decreased significantly when the cells were cultured in the riboflavin deficient media (Fig. 1c), indicating that riboflavin deficiency was successfully developed.

### Changes of proteomic profiles after riboflavin deficiency

**Figure 2:**
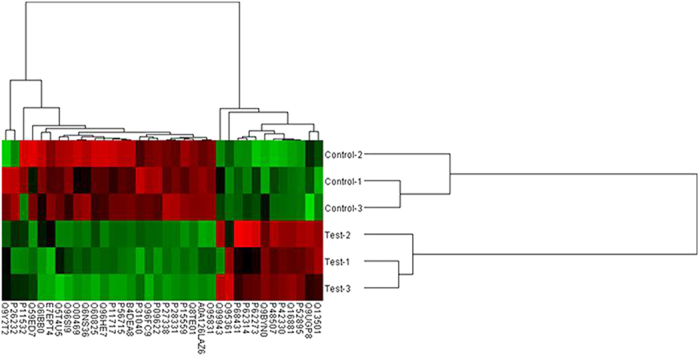
Hierarchical cluster of proteins differentially expressed between riboflavin deficient (test 1–3) and riboflavin adequate (control 1–3) samples with an FDR < 1% identified by MaxQuant. Red, high expression; green, low expression. Two main clusters of proteins can be observed, one up-regulated (right) and other down-regulated (left) in riboflavin deficient group. Protein position in the cluster can be found in Table 1.

Of the 3730 proteins covered in the shotgun proteomic analysis, 2745 proteins were identified with 99% confidence in both groups. However, a total of 85 proteins were detected only in the riboflavin deficient group and 275 proteins only in the riboflavin adequate group. Gene ontology analysis revealed that these 360 proteins unique in one of the two groups were mainly involved in the biological processes of phosphate metabolic process, chordate embryonic development and protein phosphorylation. Compared with the cells cultured in the riboflavin adequate medium, 37 proteins displayed statistically significant changes in expression (±2 fold, p < 0.05) in the riboflavin deficient group, in which 13 proteins were up-regulated and 24 down-regulated with a magnitude of fold change ranging from −6.87 to 5.43 (Fig. 2, Table 1). The top 5 proteins that were up-regulated most in expression after riboflavin deficiency were H31, RPS29, AK1C2, GSH0 and TRXR1, whereas AOFB, DLD, NQO1, NDUFS1 and DDX11 were the top 5 proteins that were down-regulated most (Table 1). These differentially expressed proteins were involved in several important biological processes as follows: 95.4% were associated with metabolic process, 47.2% with the oxidation-reduction process, and 30.6% with the electron export (Table 1). In addition, the molecular function of these proteins is mainly associated with oxidoreductase activity, flavin adenine dinucleotide binding, and cofactor binding. The cellular locations of these proteins are mainly in the organelle, endoplasmic reticulum (ER) membrane and respiratory chain.

### Pathway analysis

**Figure 3:**
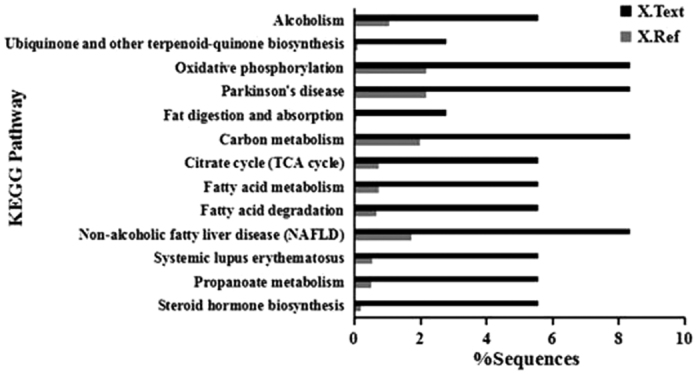
KEGG Pathway analysis. KEGG pathway enrichment analyses were applied based on the Fisher’ exact test, considering the whole quantified protein/phosphorproteins annotation as background dataset. Benjamini- Hochberg correction for multiple testing was further applied to adjust derived p-values. Only pathways with p-values under a threshold of 0.05 were considered as significant.

To further explore the impact of differentially expressed protein/phosphorproteins in cell physiological process and discover internal relations between differentially expressed proteins, enrichment analysis was performed. The pathway analysis by KEGG on differentially expressed proteins suggested that the significantly affected pathways were steroid hormone biosynthesis, propanoate metabolism, systemic lupus erythematosus, non-alcoholic fatty liver disease (NAFLD), fatty acid degradation, fatty acid metabolism, citrate cycle (TCA cycle), carbon metabolism, fat digestion and absorption, Parkinson’s disease (PD), oxidative phosphorylation, ubiquinone and other terpenoid-quinone biosynthesis, alcoholism (Fig. 3).

### Protein interaction network analysis

**Figure 4:**
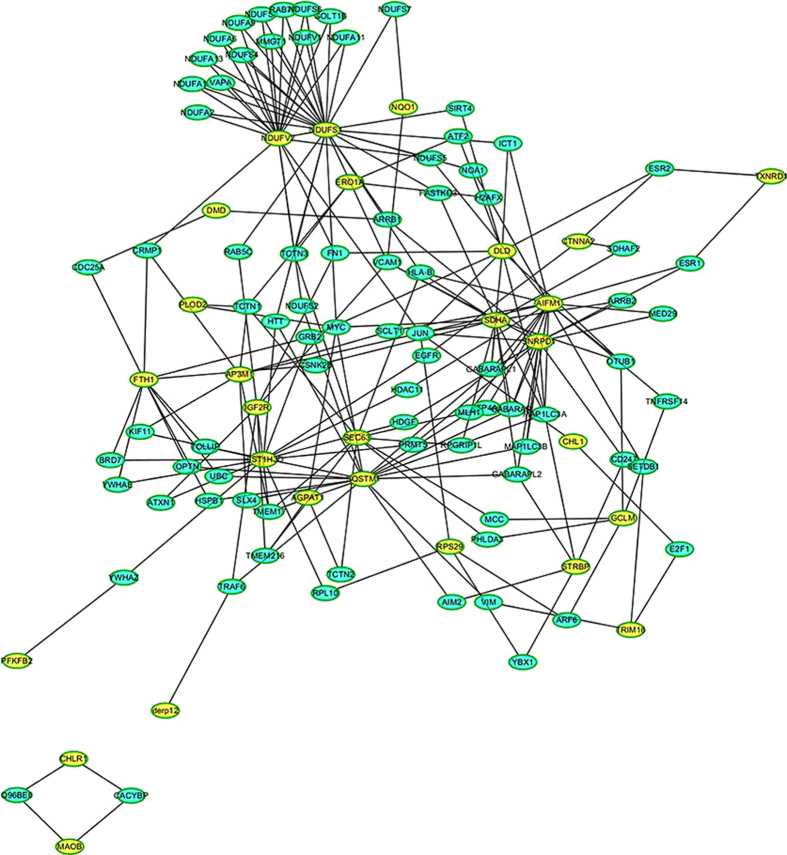
Protein–protein interaction analysis performed by InAct. Differentially expressed proteins are highlighted in yellow.

To better understand how riboflavin deficiency might affect the protein expression profiles in HepG2 cells, a protein−protein interaction network was generated. As shown in Fig. 4, the network consists of a complex interconnected web with a number of differentially expressed proteins present at key hubs, and some of them function in a variety of cellular processes (Table 1). The most abundant subnetwork is involved in energy metabolism mediated by NADH-ubiquinone oxidoreductase (NDUFS1) and NADH dehydrogenase (NDUFV2), which interact with each other. In addition, numerous proteins in mitochondrial complex I interact with NDUFS1 and NDUFV2. Succinate dehydrogenase (SDHA), which is associated with 14 under-represented proteins, is also involved in energy metabolism. Another protein with extensive connections is sequestosome-1 (SQSTM1), which interacts with 18 proteins, might play a critical role in apoptosis process.

### Immunoblotting validation of the differentially expressed proteins

**Figure 5:**
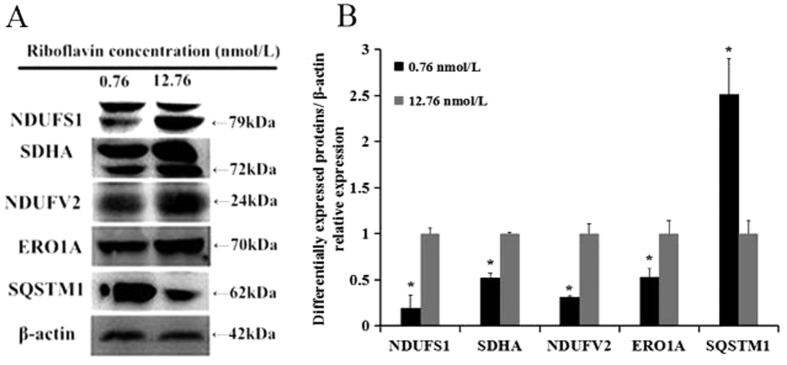
Immunoblotting validation of differentially expressed proteins. (**A**) NDUFS1, SDHA, NDUFV2, ERO1A and SQSTM1 were selected to be validated by immunoblotting test (β-actin as the control). (**B**) quantification of immunoreactive band density measured in Panel A. Data are presented as the percent change relative to control (12.76 nmol/L) samples. The blots/gels were cropped and full-length blots/gels are presented in Supplementary Fig. S1. Data are presented as mean fold values ± SD of the changed expression at the protein level. *p < 0.01.

The protein expression levels of NDUFS1, NDUFV2, SQSTM1, SDHA, and ERO1A were further analyzed by immunoblotting analysis. The results showed that the expressions of NDUFS1, NDUFV2, SDHA and ERO1A were significantly reduced, while the expression of SQSTM1 was significantly increased in the riboflavin deficient group (Fig. 5). This is consistent with the data obtained from the proteomic analysis.

## Discussion

The riboflavin deficient rat models have been frequently used in previous studies to investigate biological actions of riboflavin^14^,^15^,^16^. However, limited data are available currently concerning the molecular mechanisms behind the abnormalities induced by riboflavin deficiency. In this study, we are committed to explore the effects of riboflavin deficiency *in vitro* by a comparative label-free LC–MS/MS technique, aiming to provide useful information at the level of protein expression. The cell viability and apoptosis tests showed that riboflavin was vital in maintaining the cytoactivity of HepG2 cells. A deficient status was successfully induced when the concentration of riboflavin in the medium was lower than 12.76 nmol/L. Thus, the concentration of 0.76 nmol/L was chosen as a severely deficient level compared to the adequate level (12.76 nmol/L) in the next proteomic analysis.

Previously, several studies had demonstrated that the expression of some important proteins, as well as the activities of certain enzymes was significantly changed after riboflavin deficiency based on immunoblotting analysis or biochemical tests^17^,^18^. In the present study, we made use of a highly sensitive proteomic tool to expand the list of proteins playing specific roles in response to riboflavin deficiency. Using stringent criteria of quantitation at the peptide level, we were able to confidently detect minor variations in protein abundance in the cells. The results showed that riboflavin deficiency significantly altered the proteomic profiles in HepG2 cells. A total of 37 proteins exhibited more than 2-fold change at the protein expression level after riboflavin deficiency. Gene ontology categorization analysis indicated that many proteins involved in the electron transport and oxidation-reduction process were down-regulated in expression after riboflavin deficiency, including AOFB, DLD, NQO1, NDUFS1, AK1C2, AIFM1, SDHA, TRXR1, AK1C3, DERP12, MCAD, PLOD2, SRXN1, PLCA, NDUFV2, and ERO1A. It is noted that some of these proteins are flavin-containing enzymes, such as AOFB, DLD, NQO1, AIFM1, SDHA, TRXR1, MCAD, NDUFV2, and ERO1A^19^. Since riboflavin deficiency results in low FMN and FAD contents in the cells, it is plausible that the activities of the flavin-containing enzymes are decreased simultaneously^20^. However, the results of this studyindicate that the alterations caused by riboflavin deficiency lie not only at the enzymatic activity level, but also at the protein expression level for the flavin-containing enzymes.

It is well known that riboflavin plays an important role in the energy production^1^,^2^,^10^. In this study, we found that the protein level of medium-chain acyl-CoA dehydrogenase that catalyzes the initial step of mitochondrial fatty acid β-oxidation was down-regulated in expression by riboflavin deficiency. A significant down-regulation of several other enzymes related to energy metabolism was also confirmed at the protein level in riboflavin deficient cells, such as DLD, SDHA, NDUFS1, and NDUFV2. Protein interaction network analysis also revealed that the subnetwork mediated by NDUFS1and NDUFV2 in energy metabolism was affected significantly after riboflavin deficiency. Thereby, it is not surprising that the capacity of energy production is severely reduced after riboflavin deficiency^1^,^2^,^10^. This may contributes partly to the growth retardation in children and adolescents with low riboflavin intake. NDUFV2 and NDUFS1 are nuclear-encoded complex I subunits, involving in mitochondrial oxidative phosphorylation^21^. In the process of oxidative phosphorylation, complex I (NADH quinone oxidoreductase) acts as the entry point for electrons from the mitochondrial matrix into the electron transport chain (ETC) by catalyzing the electron transfer from NADH into the ETC subunits^22^. Mitochondrial complex I is considered one of the primary sources of ROS and increased oxidative stress resulting from ROS production is one of the proposed mechanisms for the death of dopaminergic neurons in PD^23^. Mounting evidence suggests that the impairment of mitochondrial respiratory chain, in particular complex I deficiency and the subsequent increase in ROS production may directly or indirectly contribute to the pathology of sporadic PD^23^,^24^. For example, it was reported that the variation of NDUFV2 could lead to altered energy production and mitochondrial function, which is a risk factor in the development of PD^25^. The study conducted by Keeney *et al*.^26^ showed that the core subunit NDUFS1 of complex I was oxidatively damaged in PD brains, resulting in complex I misassembling and functional impairment. Therefore, riboflavin deficiency may increase the risk of PD via down-regulation of mitochondrial complex I function. In addition, it was demonstrated that glutathione depletion was one of the earliest oxidative signs detected in the course of PD, suggesting that oxidative damage occurs even before complex I deficiency^27^. Since FAD is a coenzyme for glutathione reductase, it is not surprising that riboflavin deficiency will lead to glutathione depletion. The pathway analysis in this study also demonstrated that PD was one of the most significantly altered pathways after riboflavin deficiency. Therefore, we speculate that riboflavin may play a critical role in the physiopathologic process of PD. Further studies are warranted to investigate the relationship between riboflavin status and PD.

We also found that some differentially expressed proteins, such as AK1C2 and AK1C3, were involved in lipid metabolism. The aldo-keto reductases (AKRs) encompass a large superfamily of NAD(P)(H)-dependent oxidoreductases^28^. Members of the AK1C family, belonging to AKRs superfamily, have been suggested to play a pivotal role in maintaining steroid homeostasis^29^. There are at least four human AK1C isoforms. Among these four isoforms, AK1C2 and AK1C3 share 87.9% amino acid sequence identity and both enzymes possess 3-hydroxysteroid dehydrogenase (3-HSD) activity and catalyze 5-dihydrotesterone reduction to produce 5-androstane-3,17-diol (3-diol)^30^. Additionally, AK1C3 possesses 17-HSD activity and converts Δ^4^-androstene-3,17-dione (Δ^4^-dione) to testosterone, as well as 3, 17-diol to androsterone^31^. In addition to androgen metabolism, AK1C2 and AK1C3 also mediate prostaglandins biosynthesis^28^. In the present study, more than a two-fold increase in expression has been demonstrated for these two proteins in riboflavin deficient cells, suggesting that steroid catabolism and androgen synthesis are possibly enhanced after riboflavin deficiency. Since increased prostaglandins accumulation had been associated with prostate cancer advancement and elevated expression levels of AK1C2 and AK1C3 had been observed in the prostate tissues and bone marrow^32^,^33^,^34^, it is worthy to further investigate the effects of riboflavin deficiency on the risk of prostate cancer. Interestingly, the phospholipid metabolism is also possibly altered by riboflavin deficiency, because PLCA, an enzyme catalyzing the acylation of lysophosphatidic acid to form phosphatidic acid^35^, was up-regulated at the protein expression level in this study.

In the present study, the expression of two proteins, SEC63 and ERO1A, involved in ER stress, were found to be expressed differentially after riboflavin deficiency. SEC63 is involved in the post-translational processing of secretary proteins^36^,^37^,^38^, and acted as a component of the protein translocation machinery in the ER^39^,^40^. It is up-regulated by ER stress, and plays an important negative feedback role in the mechanism of unfolded protein response^41^. ERO1A is a FAD-dependent enzyme catalyzing the oxidative folding (formation of disulfide bonds) of secretary proteins in the ER^42^,^43^. Theoretically, the accumulation of unfolded proteins in the ER can cause ER stress and lead to unfolded protein response^44^. In this study, ERO1A was down-regulated, whereas the expression of SEC63 was up-regulated at the protein level in response to riboflavin deficiency, suggesting that riboflavin deficiency impairs the oxidative folding and subsequent secretion of proteins in HepG2 cells. Consequently, ER stress is induced by riboflavin deficiency. Manthey *et al*. demonstrated that the secretion of apolipoprotein B-100, a protein needed to be folded oxidatively in the ER, was reduced in HepG2 cells cultured in riboflavin deficient medium^45^. Since apolipoprotein B-100 is a component of lipoproteins and plays an important role in lipid metabolism, it remains to be determined whether riboflavin deficiency affects lipid transportation *in vivo*.

A significant interaction between riboflavin status and iron metabolism in relation to anemia risk was observed^1^,^10^. However, there still exist some discrepancies. Fairweather-Tait *et al*.^46^ reported that increasing hemoglobin concentration without significant change in iron absorption was found after riboflavin supplementation in the riboflavin deficient subjects. They considered that riboflavin deficiency interfered with iron utilization but not absorption^46^. However, the results of animal studies showed that riboflavin deficiency was accompanied with decreased iron absorption and increased iron loss^47^. In the present study, it is noted that the expression of ferritin (FTH1), a protein functioning in maintaining iron homeostasis, was significantly decreased at the protein level, indicating that riboflavin deficiency can impact significantly on iron absorption and utilization^48^,^49^. Powers *et al*.^5^ also demonstrated that riboflavin alone without iron supplementation was effective in improving hematologic status in young women in the United Kingdom. Therefore, riboflavin is one of the determinants in the prevention of anemia.

SQSTM1 is known as an ubiquitin-binding protein and promotes the aggregation of cullin3-modified caspase-8 within p62-dependent foci, leading to cell apoptosis^50^. A previous study showed that the transcription of SQSTM1 was increased in the case of ER stress^51^. We also found that SQSTM1 was significantly up-regulated by riboflavin deficiency in this study. This is consistent with the results we obtained from the apoptosis test, showing that the apoptosis rate was increased after riboflavin deficiency. The expression of several proteins related to cell proliferation or differentiation, such as DDX11, RP1 and IFITM2, were also altered by riboflavin deficiency, which may be related to the changes in the cell viability we found in this study. Other proteins, such as H31, PZP and F262, were differentially expressed between the two groups. Currently, we are not able to explain these changes in detail, because limited data are available or the functions of these proteins are not well elucidated.

In summary, we demonstrate for the first time that riboflavin deficiency significantly alters the protein expression profile in HepG2 cells. Many proteins involved in energy and lipid metabolic process are down-regulated remarkably in response to riboflavin deficiency. Several proteins involved in ER stress and apoptosis are also altered in expression by riboflavin deficiency. The changes of the protein expression levels of NDUFS1, NDUFV2, SQSTM1, SDHA, and ERO1A are further validated by immunoblotting analysis. More animal and human studies should be carried out to confirm these changes and elucidate the potential connection between protein expression and related outcomes after riboflavin deficiency.

## Materials and Methods

### Preparation of the riboflavin deficient medium

The riboflavin deficient medium used in this study consisted of the following components: (1) 90% (by volume) riboflavin-free customized type B medium (three portions of α-MEM and one portion of Waymouth’s MB 752/1) developed by Adeli *et al*.^52^; (2) 10% riboflavin-depleted fetal bovine serum (FBS), which was prepared by 30 min ultraviolet radiation^53^ and contained 7.6 nmol/L riboflavin as determined by a high performance liquid chromatography procedure^54^. The final concentration of riboflavin in the culture medium was 0.76 nmol/L as calculated.

### Measurement of cell viability, apoptosis and glutathione reductase activity

HepG2 cells (purchased from Shanghai Institutes for Biological Sciences, Shanghai, China) were cultured in type B medium containing 12.76 nmol/L riboflavin before being transferred into the media with different riboflavin concentrations (0.76, 3.76, 12.76, 24.76, 48.76 nmol/L). After 96 h culture, cells were harvested and the viability was determined by a MTT (methylthiazolyldiphenyl-tetrazolium bromide) colorimetric procedure^55^ and apoptosis by a flow cytometric method^56^. The glutathione reductase activity was measured by a reagent kit purchased from Nanjing Jiancheng Biotechnology Institute. The concentrations of riboflavin in the media were designed based on the studies conducted previously by Camporeale, *et al*.^43^ and Manthey, *et al*.^13^. The concentration of 0.76 nmol/L was defined as severely deficient, which represents the plasma concentration of riboflavin occurring in the preterm infants treated with phototherapy^57^ and in severely deficient patients with cystic fibrosis^58^. The concentration of 3.76 nmol/L was treated as moderate deficient, which is close to the plasma riboflavin concentration observed in the moderately deficient pregnant women^59^. The concentration of 6.76 nmol/L was considered as light deficient, which is similar to the plasma riboflavin concentration in riboflavin deficient-recovering adults^60^. The concentrations of 12.76 nmol/L, 24.76 nmol/L, and 48.76 nmol/L were treated as adequate, sufficient, and very sufficient, respectively^60^.

### Proteomic analysis and immunoblotting validation

After 96 h culture, HepG2 cells were harvested from the 0.76 nmol/L and 12.76 nmol/L groups. Total protein of the cells was extracted and reduced in the presence of 100 mmol/L DTT for 5 min on the boiling water bath, and subsequently alkylated with 50 μmol/L iodoacetamide for 30 min at room temperature in the dark. The resulting modified proteins were then digested with 40 μL sequencing grade trypsin solution (0.05 μg/μl) for 16 h at 37 °C. The peptides of each sample were desalted on C18 Cartridges (Empore™ SPE Cartridges C18, bed I.D. 7 mm, volume 3 ml, Sigma), concentrated by vacuum centrifugation and reconstituted in 40 μl of 0.1% (v/v) formic acid. The peptide content was estimated by UV light spectral density at 280 nm using an extinctions coefficient of 1.1 of 0.1% (g/l) solution that was calculated on the basis of the frequency of tryptophan and tyrosine in vertebrate proteins.

The peptide mixture was loaded onto a reverse phase trap column (Thermo Scientific Acclaim PepMap100, 100 μm × 2 cm, nano Viper C18) connected to the C18-reversed phase analytical column (Thermo Scientific Easy Column, 10 cm long, 75 μm inner diameter, 3 μm resin) in buffer A (0.1%formic acid) and separated with a linear gradient of buffer B (84% acetonitrile and 0.1% formic acid) at a flow rate of 300 nl/min controlled by IntelliFlow technology. The gradient varied from 0–45% solvent B in 100 min and 100% during 108–120 min. Finally, the gradient changed from 55–90% solvent B in 10 min and a finishing step in 4% solvent B for 10 min. Spectra scans were acquired in Q-Exactive™ mass spectrometry instrument (Thermo Scientific, USA), coupled to a nano UHPLC system via a nanoelectrospray ion source. MS data was acquired using a data-dependent top20 method dynamically choosing the most abundant precursor ions from the survey scan (300–1800 m/z) for HCD fragmentation. Automatic gain control target was set to 1e3, and maximum inject time to 50 ms. Dynamic exclusion duration was 60 s. Survey scans were acquired at a resolution of 70,000 at m/z 200 and resolution for HCD spectra was set to 17,500 at m/z 200, and isolation width was 2 m/z. Normalized collision energy was 30 eV and the underfill ratio, which specifies the minimum percentage of the target value likely to be reached at maximum fill time, was defined as 0.1%. The instrument was run with peptide recognition mode enabled.

In order to validate the results of the proteomic analysis, the protein expression levels of NDUFS1, NDUFV2, SQSTM1, SDHA, and ERO1A were further determined by the immunoblottting method. The antibodies used in the test were purchased from Abcam Company, USA. The detailed characteristics of these antibodies are presented as Supplementary Information.

### Statistical analysis

The MS data were analyzed using MaxQuant software (version 1.3.0.5.) (Max Planck Institute of Biochemistry, Germany) against the UniprotKB^61^ human database (163652 total entries, downloaded 27/05/15). Bioinformatics analysis, including hierarchical cluster, gene ontology, pathway, and network, was performed using Blast2GO (Version 3.5.0), the online Kyoto Encyclopedia of Genes and Genomes (KEGG) database (http://geneontology.org/) and the InAct molecular interaction database (http://www.ebi.ac.uk/intact/). Differentially expressed proteins between the two groups were screened using fold change method (±2 fold). Statistical significance was analyzed using Student’s t test with a value of p < 0.05 considered to be statistically significant.

## Additional Information

**How to cite this article**: Xin, Z. *et al*. Riboflavin deficiency induces a significant change in proteomic profiles in HepG2 cells. *Sci. Rep.* **7**, 45861; doi: 10.1038/srep45861 (2017).

**Publisher's note:** Springer Nature remains neutral with regard to jurisdictional claims in published maps and institutional affiliations.

## Supplementary Material

Supplementary Information

## Figures and Tables

**Table 1:** Differentially expressed proteins in HepG2 cells after riboflavin deficiency.

UniProtKB ID	Protein name	Protein description	Fold change	p-value
**electron transport**				
P27338	AOFB	Amine oxidase [flavin-containing] B	−6.87	0.0060
P09622	DLD	Dihydrolipoyl dehydrogenase, mitochondrial	−6.30	0.0008
P15559	NQO1	NAD(P)H dehydrogenase [quinone] 1	−5.29	0.0004
P28331	NDUFS1	NADH-ubiquinone oxidoreductase 75 kDa subunit, mitochondrial	−5.08	0.0004
P52895	AK1C2	Aldo-keto reductase family 1 member C2	2.90	0.0003
O95831	AIFM1	Apoptosis-inducing factor 1, mitochondrial	−2.82	0.0004
Q59ED7	Q59ED7	Putative uncharacterized protein	−2.82	0.0491
P31040	SDHA	Succinate dehydrogenase [ubiquinone] flavoprotein subunit, mitochondrial	−2.63	0.0054
Q16881	TRXR1	Thioredoxin reductase 1, cytoplasmic	2.48	0.0005
P42330	AK1C3	Aldo-keto reductase family 1 member C3	2.36	0.0011
B4DEA8	cDNA FLJ56425	Highly similar to Very-long-chain specific acyl-CoA dehydrogenase, mitochondrial	−2.10	0.0015
**oxidation-reduction process**				
P27338	AOFB	Amine oxidase [flavin-containing] B	−6.87	0.0060
P09622	DLD	Dihydrolipoyl dehydrogenase, mitochondrial	−6.30	0.0008
P28331	NDUFS1	NADH-ubiquinone oxidoreductase 75 kDa subunit, mitochondrial	−5.08	0.0004
Q8TE01	DERP12	Dermal papilla derived protein 12	−3.29	0.0001
P52895	AK1C2	Aldo-keto reductase family 1 member C2	2.90	0.0003
O95831	AIFM1	Apoptosis-inducing factor 1, mitochondrial	−2.82	0.0004
Q59ED7	Q59ED7	Putative uncharacterized protein	−2.82	0.0491
P31040	SDHA	Succinate dehydrogenase [ubiquinone] flavoprotein subunit, mitochondrial	−2.63	0.0054
Q5T4U5	MCAD	Acyl-Coenzyme A dehydrogenase, C-4 to C-12 straight chain, isoform CRA_a	−2.58	0.0324
Q16881	TRXR1	Thioredoxin reductase 1, cytoplasmic	2.48	0.0005
O00469	PLOD2	Procollagen-lysine,2-oxoglutarate 5-dioxygenase 2	−2.41	0.0144
P42330	AK1C3	Aldo-keto reductase family 1 member C3	2.36	0.0011
Q9BYN0	SRXN1	Sulfiredoxin-1	2.27	0.0100
Q99943	PLCA	1-acyl-sn-glycerol-3-phosphate acyltransferase alpha	2.26	0.0184
B4DEA8	cDNA FLJ56425	highly similar to very-long-chain specific acyl-CoA dehydrogenase, mitochondrial	−2.10	0.0015
E7EPT4	NDUFV2	NADH dehydrogenase [ubiquinone] flavoprotein 2, mitochondrial	−2.07	0.0229
Q96HE7	ERO1A	ERO1-like protein alpha	−2.01	0.0066
**lipid metabolic process**				
P09622	DLD	Dihydrolipoyl dehydrogenase, mitochondrial	−6.30	0.0008
P52895	AK1C2	Aldo-keto reductase family 1 member C2	2.90	0.0003
Q5T4U5	MCAD	Acyl-Coenzyme A dehydrogenase, C-4 to C-12 straight chain, isoform CRA_a	−2.58	0.0324
Q16881	TRXR1	Thioredoxin reductase 1, cytoplasmic	2.48	0.0005
P42330	AK1C3	Aldo-keto reductase family 1 member C3	2.36	0.0011
Q99943	PLCA	1-acyl-sn-glycerol-3-phosphate acyltransferase alpha	2.26	0.0184
B4DEA8	cDNA FLJ56425	highly similar to very-long-chain specific acyl-CoA dehydrogenase, mitochondrial	−2.10	0.0015
**response to endoplasmic reticulum stress**				
Q96FC9	DDX11	Probable ATP-dependent DNA helicase DDX11	−4.25	0.0024
B4DEA8	cDNA FLJ56425	highly similar to very-long-chain specific acyl-CoA dehydrogenase, mitochondrial	−2.10	0.0015
Q9UGP8	SEC63	Translocation protein SEC63 homolog	2.10	0.0262
Q96HE7	ERO1A	ERO1-like protein alpha	−2.01	0.0066
**response to oxidative stress**				
P52895	AK1C2	Aldo-keto reductase family 1 member C2	2.90	0.0003
P48507	GSH0	Glutamate-cysteine ligase regulatory subunit	2.58	0.0053
P42330	AK1C3	Aldo-keto reductase family 1 member C3	2.36	0.0011
Q9BYN0	SRXN1	Sulfiredoxin-1	2.27	0.0100
**tricarboxylic acid cycle**				
P09622	DLD	Dihydrolipoyl dehydrogenase, mitochondrial	−6.30	0.0008
P31040	SDHA	Succinate dehydrogenase [ubiquinone] flavoprotein subunit, mitochondrial	−2.63	0.0054
**oxidative phosphorylation**				
P28331	NDUFS1	NADH-ubiquinone oxidoreductase 75 kDa subunit, mitochondrial	−5.08	0.0004
P31040	SDHA	Succinate dehydrogenase [ubiquinone] flavoprotein subunit, mitochondrial	−2.63	0.0054
E7EPT4	NDUFV2	NADH dehydrogenase [ubiquinone] flavoprotein 2, mitochondrial	−2.07	0.0229
**cell proliferation**				
Q96FC9	DDX11	Probable ATP-dependent DNA helicase DDX11	−4.25	0.0265
P52895	AK1C2	Aldo-keto reductase family 1 member C2	2.90	0.0003
Q6NS36	FTH1	Ferritin	−2.60	0.0121
P42330	AK1C3	Aldo-keto reductase family 1 member C3	2.36	0.0011
P56715	RP1	Oxygen-regulated protein 1	−2.31	0.0018
Q6IBB0	IFITM2	IFITM2 protein	−2.05	0.0265
**cell differentiation**				
P11532	DMD	Dystrophin	−3.73	0.0297
P26232	CTNA2	Catenin alpha-2	−2.98	0.0049
P52895	AK1C2	Aldo-keto reductase family 1 member C2	2.90	0.0003
O95831	AIFM1	Apoptosis-inducing factor 1, mitochondrial	−2.82	0.0004
P42330	AK1C3	Aldo-keto reductase family 1 member C3	2.36	0.0011
P56715	RP1	Oxygen-regulated protein 1	−2.31	0.0018
O95361	TRI16	Tripartite motif-containing protein 16	2.21	0.0382
Q6IBB0	IFITM2	IFITM2 protein	−2.05	0.0265
**apoptotic process**				
P28331	NDUFS1	NADH-ubiquinone oxidoreductase 75 kDa subunit, mitochondrial	−5.08	0.0004
P52895	AK1C2	Aldo-keto reductase family 1 member C2	2.90	0.0003
O95831	AIFM1	Apoptosis-inducing factor 1, mitochondrial	−2.82	0.0004
Q13501	SQSTM1	Sequestosome-1	2.39	0.0043
P42330	AK1C3	Aldo-keto reductase family 1 member C3	2.36	0.0011
**chaperone mediated protein folding requiring cofactor**				
Q96HE7	ERO1A	ERO1-like protein alpha	−2.01	0.0066
**dUTP metabolic process**				
A0A126LAZ6	U45	U45	−2.21	0.0008
**Others**				
P68431	H31	Histone H3.1	5.43	0.0420
Q96SI9	STRBP	Spermatid perinuclear RNA-binding protein	−3.96	0.0070
P62273	RPS29	40 S ribosomal protein S29	2.95	0.0364
P20742	PZP	Pregnancy zone protein	−2.78	0.0028
P11717	IGF2R	Cation-independent mannose-6-phosphate receptor	−2.34	0.0083
P62314	SMD1	Small nuclear ribonucleoprotein Sm D1	2.26	0.0419
O60825	F262	6-phosphofructo-2-kinase/fructose-2,6-bisphosphatase 2	−2.20	0.0124
Q9Y2T2	AP3M1	AP-3 complex subunit mu-1	−2.05	0.0267
