# Supplementary material for: Riboflavin deficiency induces a significant change in proteomic profiles in HepG2 cells
Source: Sci Rep. 2017 Apr 3;7:45861. doi: 10.1038/srep45861 (PMC5377456; doi:10.1038/srep45861)
Supplement: Supplementary Information [file srep45861-s1.pdf]

Riboflavin deficiency induces a significant change in proteomic profiles in HepG2 cells

Zhonghao Xin[1,2], Lingling Pu[1], Weina Gao[1], Yawen Wang[1,2], Jingyu Wei[1], Tala Shi[1], Zhanxin Yao[1] & Changjiang Guo[*1]

[1]Institute of Health and Environmental Medicine, Tianjin, 300050, China.

[2]School of Public Health, Guangxi Medical University, Nanning, 530021, China.

Correspondence and requests for materials should be addressed to G.CJ. (email: guocjtj@126.com)

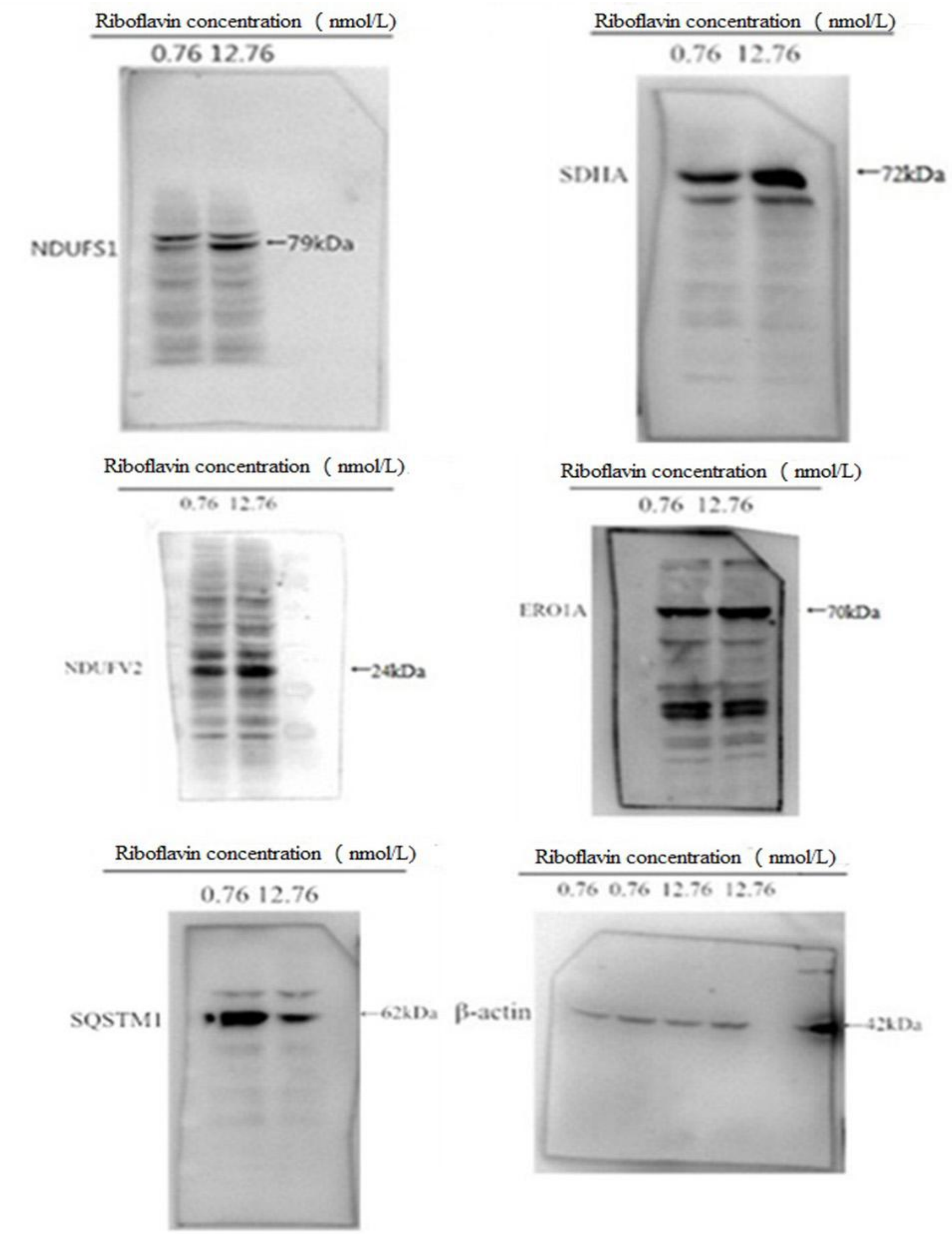

Supplementary Figure S1. Full-length blots/gels of immunoblotting validation of differentially expressed proteins.

Supplementary detailed characterization of antibodies

The primary antibodies used included the following: rabbit monoclonal anti-Ndufs1 antibody [EPR11521(B)] (1: 5000; Reacts with: mouse, rat, human; Abcam, USA), rabbit monoclonal anti-SDHA antibody [EPR9043(B)] (1: 2000; Reacts with: mouse, rat, human; Abcam, USA), rabbit monoclonal anti-NDUFV2 antibody [EPR15350(B)] (1: 5000; Reacts with: mouse, rat, human; Abcam, USA), rabbit monoclonal anti-ERO1L antibody [EPR12475(B)] (1: 5000; Reacts with: mouse, rat, human; Abcam, USA), rabbit monoclonal anti-SQSTM1 / p62 antibody [EPR4844] - Autophagosome Marker (1: 5000; Reacts with: mouse, rat, human; Abcam, USA) and mouse monoclonal anti-beta Actin antibody [mAbcam 8226] (1: 2000; Reacts with: mouse, rat, rabbit, horse, chicken, cow, dog, human, pig, zebrafish, african green monkey, chinese hamster, armenian hamster; Abcam, USA). The special secondary antibodies used was Goat Anti-Rabbit IgG H&L (HRP) (1: 10000; Abcam, USA).
